# Knowledge, Attitudes, and Practices of Dental Students on Pulp Sensitivity Tests and Diagnostic Tools

**DOI:** 10.1002/jdd.13988

**Published:** 2025-07-08

**Authors:** Camila Leão de Azevedo Pereira, Lucas Alves Jural, Manuella Freire Marzullo, Eveline Salomão Portella Mariano Lima, Marcela Barauna Magno, Patrícia A. Risso

**Affiliations:** ^1^ Faculty of Dentistry Universidade Federal do Rio de Janeiro Rio de Janeiro Brazil

**Keywords:** clinical competence | dental pulp test | dental students | diagnosis | diagnostic techniques and procedures

## Abstract

**Objective:**

To investigate the knowledge of Brazilian dental students about pulp sensitivity tests (PSTs), their attitudes concerning their aptitude for performing different diagnostic tools (DTs), and how frequently these DTs are practiced in clinical scenarios involving asymptomatic teeth.

**Methods:**

In 2024, an online questionnaire with 23 questions was developed and administered, divided into four sections: (1) sample characterization, as well as the frequency of endodontic emergency care and the participant's self‐assessment of their knowledge about PSTs; (2) knowledge of PSTs (cold, heat, and electric)—K‐PST; (3) Attitudes; (4) Practices. The mean number of correct answers for K‐PST was used to assess the knowledge level. Differences in the K‐PST mean scores between demographic and academic variables were analyzed using the Mann‐Whitney and Kruskal‐Wallis tests (*p* < 0.05).

**Results:**

Among the 628 students, 51.3% considered themselves to have good knowledge of PSTs. However, the mean number of correct answers in K‐PST was 3.43 ± 1.72. The DTs with which students felt most confident were radiography, mobility testing, and cold pulp test, respectively. No significant differences in PST knowledge were found with respect to the variables analyzed. In the clinical scenarios presented, radiographs and the collection of pain history were the most frequently reported practices, whereas PSTs were less commonly utilized.

**Conclusion:**

Although students demonstrated self‐confidence regarding their PST knowledge, the mean number of correct answers indicates a low level of knowledge. Moreover, the use of these tests does not appear to be a common practice among students.

## 1 Introduction

Diagnosing the pulp condition is essential for establishing an appropriate dental treatment plan [1]. This diagnostic process is complex and relies on professional experience, proper execution, and interpretation of a sequence of steps that includes the chief complaint, medical and dental history, clinical examination, and the use of several diagnostic tools (DTs), such as diagnostic tests and radiographic exams [2].

The presence of pulp vascularization is considered the primary indicator of pulp vitality [3], and cold (CPT), heat (HPT), and electric pulp tests (EPT) have traditionally been employed [4]. More recently, new tests have been developed, such as laser Doppler flowmetry (LDF) and pulse oximetry (PO), though they remain restricted in use [4]. Furthermore, true pulp vitality can only be determined histologically, which is clinically unfeasible [3, 5]. Nevertheless, clinical diagnoses based on pulp sensitivity tests (PSTs) generally correspond with histological findings in most cases [5]. Thus, despite being subjective, PSTs can contribute to a reliable pulp diagnosis when used together with clinical and radiographic evaluation [6].

Evaluating pain plays an important role in clinical dentistry, as the presence, characteristics, or absence of pain may indicate the state of the pulp condition [7]. However, it is known that patient‐reported symptoms may lead to more invasive treatments such as root canal therapy, even if these treatments are unwarranted [8, 9]. Conversely, lack of symptoms may lead to neglect. Therefore, pulp condition evaluation is recommended for all teeth indicated for restorative treatment [10].

The challenge of diagnosing pulp conditions reflects the need to employ more than one diagnostic test. Test selection and interpretation are subjective, varying according to experience, training, and knowledge, with an evident lack of consensus on endodontic diagnosis [11]. In this regard, the ability of professionals to determine the pulp diagnosis is highly relevant. However, there is limited evidence describing diagnostic test use by dentists [11, 12] and dental students [2], and no published study has evaluated the technical knowledge of students about conducting PSTs.

During their education, it is crucial for students to stay up to date with contemporary theoretical and practical knowledge and to develop the relevant skills, since they will likely replicate the patterns and behaviors they acquire as students [2]. In order to achieve an accurate endodontic diagnosis, a variety of factors must be taken into account, with clinical training being one of the most important. Diagnostic skills [13] and self‐confidence in endodontic procedures have been shown to be low [14, 15], whereas endodontic‐related stress levels have been found to be high [16].

Measuring dental students’ knowledge of pulp tests and their application is essential for assessing diagnostic skills and providing necessary information to enhance undergraduate education. In this context, even given the clinical relevance of using PSTs, studies [2, 16] have shown that undergraduate students may have limited training and low confidence in taking the tests. This educational gap can impact the academic training and performance of future professionals. Therefore, the objective of this study was to investigate the knowledge of dental students about PSTs, their attitudes related to aptitude for performing different DTs, and the frequency of practices and use of those tests in clinical scenarios involving asymptomatic teeth.

## 2 Methods

### 2.1 Study Design and Participants

This cross‐sectional study was previously approved by the local research ethics committee (approval no. 4.904.269). The study followed STROBE guidelines [17], as well as CHERRIES [18].

Inclusion criteria consisted of Brazilian undergraduate students enrolled in the final year of their dental program, aged 18 or older. Participants who had not taken endodontics courses and/or those who did not fully answer the specific questions on knowledge about PSTs and self‐confidence in carrying out DTs were excluded. Data were collected between October 2023 and April 2024.

### 2.2 Variables Studied

#### 2.2.1 Sample Characterization

Data were collected on sex, age, geographic region of Brazil (North, Northeast, Midwest, Southeast, South), type of university (private or public), and academic term (second to last or last). Additionally, information was gathered on the frequency of handling endodontic emergencies (“never,” “rarely,” “sometimes,” “often,” or “always”) and participants’ self‐assessment regarding their knowledge of PSTs (“none,” “insufficient,” “moderate,” “good,” or “excellent”).

#### 2.2.2 Knowledge of PSTs

Nine questions (Table 1) were used to assess knowledge about performing PSTs (three statements on each type of test: cold, heat, and electric). Each statement was answered with “agree,” “disagree,” or “I don't know.” At the end of the survey, each correct answer received a score of 1, whereas incorrect or “I don't know” responses were scored 0. Thus, the total possible score on this variable ranged from 0 to 9.

**TABLE 1:** Questions on knowledge of pulp sensitivity tests and their respective scoring based on the literature.

Test	Statement	Agree	Disagree	I don't know
**Cold (Regarding cold testing using refrigerant spray)**	1. The use of isolation (relative or absolute) is mandatory for performing the test.	1	0	0
	2. The spray should be applied to a cotton pellet or cotton swab, placed on the buccal surface near the cervical portion of the tooth.	1	0	0
	3. If the test needs to be repeated, it can be repeated immediately.	0	1	0
**Heat (Regarding heat testing with a heated gutta‐percha stick)**	4. The use of isolation (relative or absolute) is mandatory for performing the test.	1	0	0
	5. Solid petroleum jelly should be applied to the coronal surface.	1	0	0
	6. The gutta‐percha stick should be applied when it is opaque.	0	1	0
**Electric**	7. The use of isolation (relative or absolute) is mandatory for performing the test.	1	0	0
	8. The tooth and electrode must be coated with toothpaste.	1	0	0
	9. The electrode should be placed on any existing restoration in the tooth, due to better conductivity.	0	1	0

#### 2.2.3 Attitudes Related to Aptitude for Performing Different DTs

Each participant self‐reported their aptitude for performing PSTs and other DTs used for pulp condition diagnosis (Table 2). For each PST or DTs investigated, participants chose among “I don't know this,” “I know it but do not feel able to perform/analyze it,” or “I know it and feel able to perform/analyze it.”

**TABLE 2:** Diagnostic tools.

Category	Item
**Pulp Sensitivity Tests**	Cold pulp test (with cold spray)
	Heat Pulp test (with heated gutta‐percha stick)
	Electric Pulp test
**Pulp Vitality Tests**	Laser Doppler flowmetry
	Pulse oximetry
**Diagnostic tools**	Cavity test
	Selective anesthesia test
	Palpation of the patient's face
	Apical palpation
	Horizontal percussion test
	Vertical percussion test
	Mobility test
	Periapical radiography
	Fistulography
	Cone‐beam computed tomography

#### 2.2.4 Practice and Use of DTs in Clinical Scenarios Involving Asymptomatic Teeth

Four clinical scenarios in asymptomatic teeth were presented: deep caries, teeth requiring direct restoration, restored teeth requiring indirect restoration, and healthy teeth requiring indirect restoration. Participants were asked how frequently they would collect pain history, and perform cold, heat, and electric sensitivity tests, the cavity test, and radiographs in these clinical situations. The response options were “never,” “rarely,” “sometimes,” “often,” or “always.” The complete questionnaire can be accessed in the supplementary file.

### 2.3 Preliminary Application and Sample Size Calculation

The 23‐question survey used in this study was validated by assessing its psychometric properties among dentists [19] and then adapted for the present research (Suppl 1). The validation process conducted by Lima ESPM in 2019 [19] included content validation by endodontics professors, referred to as “judges,” using the Content Validity Index (CVI). The final CVI was 3.8, indicating that the judges found the items relevant, representative, and operationally appropriate [20]. Subsequently, the instrument's psychometric properties were assessed in a pilot study with dentists. Convergent validity was tested by comparing knowledge scores between endodontics specialists and non‐specialists, with specialists scoring significantly higher (p < 0.001), indicating strong convergent validity. Temporal stability was evaluated through a test‐retest procedure conducted 15 days apart, yielding an intraclass correlation coefficient (ICC) of 0.8, which indicates good reliability over time. Internal consistency was assessed using Cronbach's alpha and McDonald's omega, both of which were 0.7, supporting the instrument's reliability [21].

To conduct the current study, the instrument was presented to a pilot sample of the target population, consisting of 12 students from different educational institutions (one public and one private), who confirmed the clarity and comprehensibility of the items. Subsequently, the instrument was preliminarily answered by 43 students, and the mean number of correct responses regarding PST knowledge was used to calculate the sample size required for this study.

For the sample calculation of a finite population of 26,664—according to the number of new dentist registrations in the Brazilian Federal Council of Dentistry in 2022 (https://website.cfo.org.br/dados‐estatisticos‐de‐profissionais‐e‐entidades‐ativas‐por‐ano/), two main outcomes were considered:

1. **Knowledge of PSTs**: Based on the 3.26 ± 1.2 mean correct score observed among 43 students from the pilot study, and with 43 out of 105 students (40 from a public institution and 65 from a private institution in the first half of 2023) already included, a 95% confidence level and 12% margin of error were adopted (https://pt.surveymonkey.com/mp/margin‐of‐error‐calculator/). Using the 26,664 figure as the finite population, the sample size was calculated to be 382 students.
2. **Use of Diagnostic Tools in Clinical Scenarios for Asymptomatic Teeth**: Frequencies of PST use/indication by dental students in different clinical scenarios, ranging between 35% and 95%, were reported by Alobaoid et al. The lowest frequency (35%) was considered to avoid bias in the sample size calculation. Adopting 35%, ∞ 5%, and the finite population of 26,664, resulted in a sample size of 346 students.

To cover both outcomes, the larger number (382) was chosen as the minimum final sample size. The USP Bauru calculator (http://estatistica.bauru.usp.br/calculoamostral/calculos.php) was used to estimate the sample size.

### 2.4 Data Collection Strategy and Implementation

The questionnaire was hosted on the SurveyMonkey platform and distributed via messaging apps and social media (WhatsApp, Instagram, Facebook).

A list of public and private dental schools nationwide was compiled using social networks. Data were then collected by “snowball sampling,” a non‐probabilistic chain referral method whereby initial participants (contacted via social media) invite other potential participants [22, 23].

### 2.5 Data Analysis

Data were tabulated and categorized, and sample characteristics were presented through frequencies, means, and standard deviations (SDs) for both predictor variables and outcomes. Parametric distribution was evaluated using the Shapiro‐Wilk test (*p* = 0.00). Differences in mean or median PST knowledge scores across demographic and academic variables were assessed with the Mann‐Whitney and Kruskal‐Wallis tests, at a 0.05 significance level. Statistical analyses were performed in SPSS version 22.0 (SPSS for Windows, version 22.0; IBM Corporation, Armonk, NY, USA).

## 3 Results

### 3.1 Main Characteristics of the Sample and PST Knowledge

**FIGURE 1:**
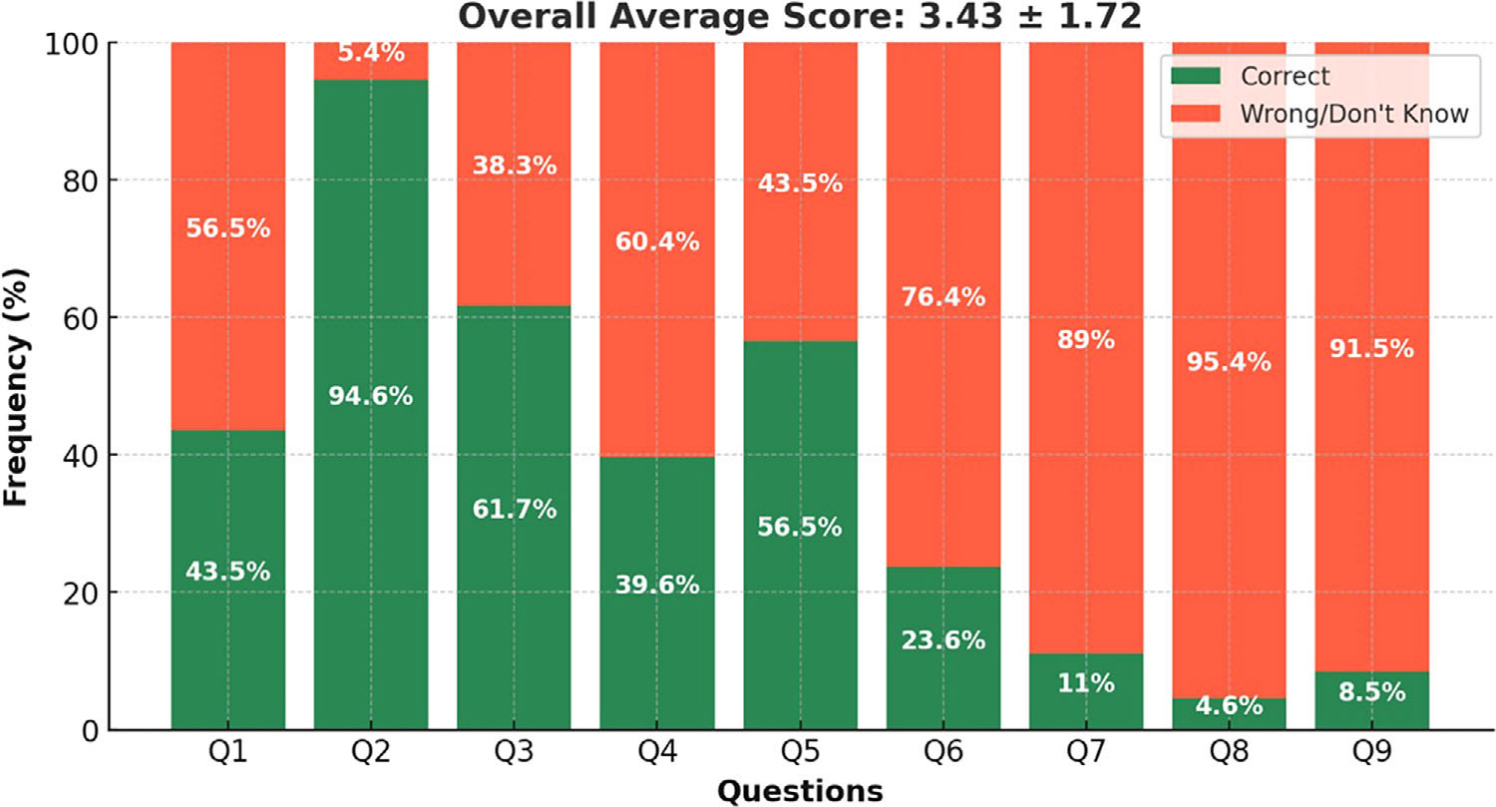
Knowledge regarding pulp sensitivity tests. Q1, Q2, Q3 – questions related to the cold pulp test. Q4, Q5, Q6 – questions related to the heat pulp test. Q7, Q8, Q9 – questions related to the electric pulp test.

A total of 628 students (mean age 23.8 ± 3.3 years) completed the questionnaire. Students who participated in the pilot testing phase were excluded from these results. The overall mean number of correct answers to the PST knowledge questions was 3.43 ± 1.72. Students scored the highest on questions about CPT, and the lowest on EPT (Figure 1).

No statistically significant differences were found in the mean PST knowledge scores across the demographic and academic variables analyzed (Table 3).

**TABLE 3:** Demographic characteristics of participants (*n* = 628), self‐assessment of PST knowledge, and performance of endodontic emergencies.

Variables	Categories (*n*; %)	Mean Knowledge Score	*p*‐value
**Sex**	Female (464; 73.9%)	3.49 ± 1.76	0.90^a^
	Male (164; 26.1%)	3.25 ± 1.59	
**Geographic regions**	North (114; 18.2%)	3.26 ± 1.72	0.63^b^
	Northeast (147; 23.4%)	3.60 ± 1.81	
	Midwest (61; 9.7%)	3.46 ± 1.76	
	Southeast (202; 32.2%)	3.44 ± 1.71	
	South (104; 16.6%)	3.35 ± 1.58	
**Type of University**	Public (309; 49.2%)	3.57 ± 1.73	0.19^a^
	Private (319; 50.8%)	3.29 ± 1.70	
**Academic term**	Second to last (341; 54.3%)	3.36 ± 1.76	0.33^a^
	Last (287; 45.7%)	3.51 ± 1.67	
**Self‐assessment of knowledge**	Insufficient (22; 3.5%)	2.77 ± 1.37	0.11^b^
	Moderate (238; 37.9%)	3.39 ± 1.63	
	Good (322; 51.3%)	3.43 ± 1.74	
	Excellent (46; 7.3%)	4.0 ± 1.88	
**Performing endodontic emergencies**	Never (51; 8.1%)	3.33 ± 1.90	0.23^b^
	Rarely (171; 27.2%)	3.43 ± 1.75	
	Sometimes (278; 44.3%)	3.42 ± 1.69	
	Often (107; 17%)	3.66 ± 1.61	
	Always (21; 3.3%)	3.67 ± 1.52	

^a^ Mann‐Whitney test

^b^ Kruskal‐Wallis test

### 3.2 Attitudes Related to Aptitude for Performing Different Diagnostic Tools

A large majority of the students reported knowing/feeling competent in periapical radiography (99.7%) and CPT (97.6%). Conversely, only 6.2% of students felt competent in EPT. Students’ self‐assessed aptitude for these DTs is shown in Table 4.

**TABLE 4:** Aptitude for performing diagnostic tools (*n* = 628).

Test/Exam	I don't know (*n*, %)	I know it, but do not feel apt (*n*, %)	I know it and feel apt (*n*, %)
Periapical radiography	0 (0)	2 (0.3)	626 (99.7)
Mobility test	4 (0.6)	7 (1.1)	617 (98.2)
Cold pulp test (spray)	1 (0.2)	14 (2.2)	613 (97.6)
Vertical percussion test	4 (0.6)	16 (2.6)	608 (96.8)
Horizontal percussion test	6 (1)	20 (3.2)	602 (95.9)
Facial palpation	11 (1.8)	48 (7.6)	569 (90.6)
Apical palpation	34 (5.4)	66 (10.5)	528 (84.1)
Fistulography	30 (4.8)	155 (24.7)	443 (70.5)
Cavity test	106 (16.8)	106 (16.8)	416 (66.3)3
Selective anesthesia test	95 (15.2)	158 (25.1)	375 (59.7)
Heat Pulp test (heated gutta‐percha stick)	38 (6.1)	268 (42.6)	322 (51,3)
Cone‐beam computed tomography	56 (8.9)	320 (51)	252 (40.1)
Pulse oximetry	426 (67.9)	155 (24.7)	46 (7.3)
Electric Pulp test	290 (46.1)	300 (47.7)	39 (6.2)
Laser Doppler flowmetry	500 (80.7)	112 (17.9)	9 (1.4)

### 3.3 Practice and Using Diagnostic Tools for Diagnosing Pulp Condition in Different Clinical Scenarios Involving Asymptomatic Teeth

Pain history collection and radiographic exams were reported as the most frequent practices across all clinical scenarios. Among PSTs, CPT was used most frequently, whereas EPT was the least used DT (Table 5).

**TABLE 5:** Frequency of using diagnostic tools for pulp condition diagnosis in different clinical scenarios involving asymptomatic teeth (*n* = 628).

In the following clinical situations involving asymptomatic teeth, how often do you perform these auxiliary diagnostic tests?			
Scenario	Never/Rarely	Sometimes	Often/Always
	(*n*, %)	(*n*, %)	(*n*, %)
**Deep caries**			
Pain history	3 (0.5%)	3 (0.5%)	622 (99%)
Cold pulp test	30 (4.8%)	52 (8.3%)	544 (86.9%)
Heat pulp test	398 (63.4%)	91 (14.5%)	139 (22.1%)
Electric Pulp test	585 (93.2%)	30 (4.8%)	13 (2.1%)
Cavity test	305 (48.6%)	117 (18.7%)	205 (32.7%)
Periapical radiograph	0 (0%)	2 (0.3%)	626 (99.7%)
**Teeth requiring direct restoration**			
Pain history	16 (2.5%)	24 (3.8%)	588 (93.6%)
Cold pulp test	154 (24.6%)	151 (24.1%)	322 (51.4%)
Heat pulp test	457 (73%)	81 (12.9%)	88 (14.1%)
Electric Pulp test	597 (95.2%)	17 (2.7%)	13 (2.1%)
Cavity test	341 (54.6%)	120 (19.2%)	164 (26.2%)
Periapical radiograph	8 (1.3%)	17 (2.7%)	603 (96%)
**Healthy teeth needing indirect restoration**			
Pain history	37 (5.9%)	36 (5.7%)	554 (88.4%)
Cold pulp test	209 (33.3%)	136 (21.7%)	282 (45%)
Heat pulp test	480 (76.6%)	65 (10.4%)	82 (13.1%)
Electric Pulp test	599 (95.8%)	16 (2.6%)	10 (1.6%)
Cavity test	397 (63.4%)	98 (15.7%)	131 (20.9%)
Periapical radiograph	23 (3.7%)	27 (4.3%)	577 (92%)
**Restored teeth needing indirect restoration**			
Pain history	28 (4.5%)	31 (4.9%)	569 (90.6%)
Cold pulp test	195 (31.2%)	130 (20.8%)	301 (48.1%)
Heat pulp test	480 (76.7%)	56 (8.9%)	90 (14.4%)
Electric Pulp test	594 (95%)	20 (3.2%)	11 (1.8%)
Cavity test	389 (62.2%)	95 (15.2%)	141 (22.6%)
Periapical radiograph	14 (2.2%)	27 (4.3%)	587 (93.5%)

## 4 Discussion

PSTs are valuable tools for clinical decision‐making in dentistry, yet their use has not been thoroughly examined among professionals or dental students. In our sample, most students believed their PST knowledge was good; however, the mean correct score of 3.43 ± 1.72 out of 9 could be considered low, given that many academic institutions use 5.0 as the minimum passing grade. This result may be linked to the fact that more than 90% of participants indicated they did not know about or did not feel capable of performing EPTs, reflecting the limited use of this tool in Brazilian dental schools. This correlates with a study in southeastern Brazil [24], which found that over 40% of participants never used EPT. Similarly, heat testing has lower accuracy compared to CPT and thus is less widely used in professional practice, contributing to the knowledge gap we observed.

The students' self‐confidence did not align with their actual knowledge, particularly regarding the execution of PSTs, which also affected their use in cases of asymptomatic teeth. Despite the clinical importance of these tests in identifying the state of the pulp and guiding emergency treatments, students often encounter the content in a fragmented manner across several disciplines. The theoretical content typically does not coincide with preclinical practical training, and clinical training is conducted under supervision, resulting in students lacking autonomy in making diagnoses. This gap in training compromises clinical competence in endodontic diagnosis, which is fundamental to general practice. This highlights two points of interest for educational practice: the need to engage students in reflective self‐assessment to foster critical thinking and the necessity for improved diagnostic instruction for final‐year dental students, potentially including structured clinical guides to bridge theoretical knowledge and practical training [25], enabling students to use the tests more routinely as they gain more knowledge.

In alignment with previous studies [26, 27], most participants in our sample were female (73.9%). Academic terms (penultimate vs. final) and institution type (public vs. private) were relatively evenly distributed, allowing a broader comparative analysis. The regional distribution mirrored the proportions of dental schools in each region, with the Southeast yielding the highest response rates [28].

No significant variation in PST knowledge was found across demographic or academic variables. Alobaoid et al. indicated that diagnostic skills do improve with advancing academic years, though a sizable percentage of final‐year students still did not feel fully competent in endodontic diagnosis. Edwards et al. showed that newly graduated dentists were less likely to use PSTs than those with postgraduate training, underscoring how diagnostic competence evolves based on education. In our study, penultimate‐term students had knowledge scores comparable to those in their final term, probably because they had already completed most of the theoretical training in endodontics. These findings highlight the necessity of curricular improvements, integrating both theory and practice from an earlier stage, given the fundamental importance of pulp diagnosis in professional dental practice.

Handling dental emergencies is common in general dentistry [29]. Nevertheless, only about 20% of our participants frequently performed such procedures, possibly due to the lack of emergency clinics at many dental schools. Such a gap may undermine students’ PST knowledge and their confidence in making rapid diagnostic and treatment decisions [14, 15, 29].

Students’ self‐perceived competency also appears linked to DT availability in their clinical training. The majority felt unprepared for LDF and PO, which are known to be effective in identifying pulp vitality, especially in diagnostic challenges like dental trauma [30, 31]. Costs and the need for specialized training often limit their routine use. Thus, it is plausible that these novel DTs have not been thoroughly integrated into dental curricula.

In contrast, percussion and palpation tests were among the techniques students felt most comfortable performing, mirroring findings from Alobaoid et al. Radiographic examinations and pain history were the most commonly used DTs, consistent with previous studies [12, 32]. The high incidence of radiographs may reflect an overemphasis on radiographic interpretation in undergraduate studies or easier access in clinical settings. Radiographic examinations are generally perceived as more objective, possibly leading students to rely on them more than on subjective methods such as PSTs. However, while important, radiographs should be interpreted alongside PSTs to enhance diagnostic accuracy, especially in asymptomatic cases. Since asymptomatic pulpitis and silent pulp necrosis can occur [33], collecting comprehensive diagnostic data helps prevent restorative procedures in teeth with undetected pulp alterations [10, 34, 35, 36].

While cold sensitivity testing was the most frequently mentioned PST, electric testing remained the least used, consistent with prior findings in both student [24] and professional [12] contexts. In our study, only asymptomatic clinical cases were presented, which might have led students to assume normal pulp status and thus not consider PSTs essential prior to treatment [37]. Tan et al. similarly found that few dentists regularly used PSTs for all patients, symptomatic or otherwise. However, asymptomatic pulpitis is roughly as common as the symptomatic form [33], and dental students must be proficient in diagnosing both [2].

Because cavity testing is invasive and irreversible [38], it is generally reserved for suspicion of necrosis. Surprisingly, 32.7% of students reported always using cavity testing in cases of deep caries, implying some might be improperly using hand instruments to explore the lesion (possibly fearing pulp exposure [16]) rather than employing burs at high or low speed [38, 39]The study has limitations common to online surveys, such as recall bias and inattentive responses. Nonetheless, the methodology provided extensive coverage of students nationwide in a large country.

The findings of this study highlight the need to enhance the teaching of endodontic DTs in dental curricula. By incorporating more structured preclinical training and simulation‐based learning, students can gain greater confidence and accuracy in applying PST. Educators should also integrate clinical reasoning exercises to help students interpret test results within a comprehensive diagnostic context. Additionally, it is essential to emphasize the use of PST alongside radiographic and other examinations, even for asymptomatic teeth requiring dental procedures. Future studies should explore the effectiveness of targeted educational interventions, such as workshops or simulation laboratories, in improving students' competence in PST. They should also evaluate the relationship between self‐confidence and actual knowledge in various pulp diagnostic scenarios and assess the teaching of diagnostic methods. Multicenter studies could determine whether these gaps are consistent across curricula.

Multiple factors—clinical experience [40], self‐confidence, and academic training—influence clinical judgment. Proper pulp diagnosis is pivotal for daily dental practice, minimizing errors in treatment planning. By examining final‐year dental students’ self‐confidence regarding PSTs, knowledge of specific tests, and their use in asymptomatic clinical scenarios, this study highlights the importance of not only enhancing curriculum design but also conducting follow‐up research to evaluate the effectiveness of targeted educational interventions and to better understand how diagnostic confidence evolves throughout clinical training.

## 5 Conclusion

Despite students’ reported self‐confidence about PSTs, the mean knowledge score was relatively low, which may have contributed to the underuse of these DTs in asymptomatic clinical scenarios. Conversely, the high percentage of students feeling competent in periapical radiography was reflected in its frequent use across the presented cases. Further investigations are recommended to better understand the factors influencing the utilization of DTs in clinical practice.
